# Acceptability of an open-label wait-listed trial design: Experiences from the PROUD PrEP study

**DOI:** 10.1371/journal.pone.0175596

**Published:** 2017-04-20

**Authors:** Mitzy Gafos, Elizabeth Brodnicki, Monica Desai, Sheena McCormack, Will Nutland, Sonali Wayal, Ellen White, Gemma Wood, Tristan Barber, Gill Bell, Amanda Clarke, David Dolling, David Dunn, Julie Fox, Lewis Haddow, Charles Lacey, Anthony Nardone, Killian Quinn, Caroline Rae, Iain Reeves, Michael Rayment, David White, Vanessa Apea, Wilbert Ayap, Claire Dewsnap, Yolanda Collaco-Moraes, Gabriel Schembri, Yinka Sowunmi, Rob Horne, on behalf of the PROUD Study Team

**Affiliations:** 1 Medical Research Council Clinical Trials Unit, Institute of Clinical Trials & Methodology, University College London, London, United Kingdom; 2 HIV/STI Department, Public Health England, London, United Kingdom; 3 Directorate of HIV and GU medicine, Chelsea and Westminster Hospital NHS Foundation Trust, London, United Kingdom; 4 Sigma research, London School of Hygiene & Tropical Medicine, London, United Kingdom; 5 Centre for Sexual Health and HIV Research, Mortimer Market Centre, London, United Kingdom; 6 Sheffield Health, Sheffield Teaching Hospitals NHS Foundation Trust, Sheffield, United Kingdom; 7 Claude Nichol Centre, Royal Sussex County Hospital, Brighton, United Kingdom; 8 Guy’s and St Thomas’ NHS Foundation Trust, London, United Kingdom; 9 Research Department of Infection and Population Health, University College London, London, United Kingdom; 10 York Hospitals NHS Foundation Trust, York, United Kingdom; 11 King’s College Hospital NHS Foundation Trust, London, United Kingdom; 12 Department of Sexual Health, Homerton University Hospital NHS Foundation Trust, London, United Kingdom; 13 Heart of England NHS Foundation Trust, Birmingham, United Kingdom; 14 Ambrose King Centre, Barts Health NHS Trust, London, United Kingdom; 15 St Mary’s Hospital, Imperial College NHS Foundation Trust, London, United Kingdom; 16 Manchester Centre for Sexual Health, Central Manchester University Hospitals NHS Foundation Trust, Manchester, United Kingdom; 17 School of Pharmacy, University College London, London, United Kingdom; University of New South Wales, AUSTRALIA

## Abstract

**Background:**

PROUD participants were randomly assigned to receive pre-exposure prophylaxis (PrEP) immediately or after a deferred period of one-year. We report on the acceptability of this open-label wait-listed trial design.

**Methods:**

Participants completed an acceptability questionnaire, which included categorical study acceptability data and free-text data on most and least liked aspects of the study. We also conducted in-depth interviews (IDI) with a purposely selected sub-sample of participants.

**Results:**

Acceptability questionnaires were completed by 76% (415/544) of participants. After controlling for age, immediate-group participants were almost twice as likely as deferred-group participants to complete the questionnaire (AOR:1.86;95%CI:1.24,2.81). In quantitative data, the majority of participants in both groups found the wait-listed design acceptable when measured by satisfaction of joining the study, intention to remain in the study, and interest in joining a subsequent study. However, three-quarters thought that the chance of being in the deferred-group might put other volunteers off joining the study. In free-text responses, data collection tools were the most frequently reported least liked aspect of the study. A fifth of deferred participants reported ‘being deferred’ as the thing they least liked about the study. However, more deferred participants disliked the data collection tools than the fact that they had to wait a year to access PrEP. Participants in the IDIs had a good understanding of the rationale for the open-label wait-listed study design. Most accepted the design but acknowledged they were, or would have been, disappointed to be randomised to the deferred group. Five of the 25 participants interviewed reported some objection to the wait-listed design.

**Conclusion:**

The quantitative and qualitative findings suggest that in an environment where PrEP was not available, the rationale for the wait-listed trial design was well understood and generally acceptable to most participants in this study.

## Introduction

Randomised placebo-controlled trials are considered the ‘gold standard’ when evaluating an investigational product for prevention. Randomised placebo-controlled trials have provided a breakthrough in HIV prevention by demonstrating the benefit of oral and topical pre-exposure prophylaxis (PrEP) [1–7]. However, results of a few placebo-controlled trials have been hindered by low levels of adherence. This has resulted in trial results either underestimating the level of protection offered by PrEP [1], or showing no benefit despite evaluating efficacious products [8–10]. Poor adherence in placebo-controlled trials initially undermined confidence in oral products for women and has potentially jeopardised the development of a topical product considered biologically efficacious against both HIV and herpes simplex virus [4, 11].

Measuring product acceptability has been central to PrEP trials, given participants are unlikely to use a product they consider unacceptable [12]. However, it is important to distinguish between product acceptability and product adherence, as it does not necessarily follow that participants are more likely to use a product they consider acceptable [13]. The Necessity-Concerns-Framework (NCF) predicts adherence by measuring an individual’s belief about the necessity of taking medication balanced against their concerns about taking it [14, 15]. This balance of necessity and concern is skewed *against* adherence in placebo-controlled trials where there is no benefit of taking a placebo, unclear benefit of taking the investigational product, and a heightened focus on potential side effects [16]. Certainly, higher risk participants have been more adherent in open label extension phases of trials than during the placebo-controlled phase [17, 18].

Although randomised placebo-controlled trials are the ‘gold standard’, when there is strong evidence of efficacy, there is growing interest in trial designs that promote instead of hinder adherence [19]. One alternative is the randomised wait-listed control trial design whereby participants either receive the intervention immediately or after a deferred period. Wait-listed trial designs have been used extensively in health research, for example in the field of mental health [20], cognitive function [21], nutrition [22], pain management [23] and organ transplantation [24]. However, open-label wait-listed trial designs have rarely been used in biomedical HIV prevention trials [25].

In 2011, a PrEP e-group, which included community members, healthcare providers, policy-makers and researchers, identified the need to understand the potential public-health benefit and cost effectiveness of PrEP for the National Health Service in England [26]. They agreed that a randomised controlled trial (RCT) was necessary but that a placebo-controlled trial would not be able to measure the net-effect of PrEP, taking account of efficacy, adherence, and any change in sexual behaviour as a result of using PrEP. Consequently, the PROUD study was designed as a randomised open-label wait-listed trial. However, given PrEP was approved for use in the United States of America, there were questions about whether the deferred start would be acceptable to participants. In this paper, we present findings from quantitative and qualitative data on the acceptability of the wait-listed trial design in the context of the acceptability of other study procedures in the PROUD study [27, 28].

## Methods

PROUD was a pragmatic open-label wait-listed RCT designed to evaluate the effectiveness of daily oral Truvada (tenofovir disoproxil fumarate and emtricitabine) as HIV pre-exposure prophylaxis (PrEP). The study was only called PROUD, although this was originally derived from the longer title of: **PR**e-exposure **O**ption for reducing HIV in the **U**K: an open-label randomisation to immediate or **D**eferred daily Truvada for HIV negative gay men. PROUD was designed as a pilot study to assess the acceptability of the study design and procedures in advance of a larger scale clinical trial. However, due to higher than anticipated HIV incidence and effectiveness of PrEP, the clinical trial objectives were met during the pilot [29].

Recruitment took place from November 2012 to April 2014 at 13 sexual health clinics in England, eight in London and five outside of London. The study was advertised at the participating sexual health clinics, during community outreach activities, online and via sexual networking applications. Eligibility criteria included being gay or a man having sex with men, being a trans women having sex with men, and being assigned a male gender at birth. Eligible participants also had to be HIV negative, aged 18 or above, to have reported condomless anal sex in the last 90 days and expected to have it again in the next 90 days, and to have previously attended the enrolling clinic. Before enrolment, the study was explained using the participant information sheet (PIS) and participants provided written informed consent. The UK Community Advisory Board (http://www.ukcab.net/) reviewed the protocol, which included recommended changes to the eligibility criteria which supported recruitment, and substantially revised the PIS and informed consent forms, which included improving the readability score from 35 (academic level) to more than 70 (general population level). At enrolment, participants were randomly assigned (1:1) to receive daily Truvada either immediately or wait-listed to receive Truvada after a deferred period of one year. Regular sexual partners were encouraged to enrol together and both partners allocated to the same group to minimise the possibility of drug sharing. The study design, baseline characteristics of the cohort and results have been presented elsewhere [27, 28].

After enrolment, participants were asked to attend the clinic quarterly for HIV and STI screening, with an extra visit one month after starting PrEP to check safety and tolerability (at month 1 for the immediate group and month 13 for the deferred group). Notifications of HIV sero-conversion for participants who did not return for follow-up visits were sourced from electronic clinic records in other PROUD clinics and Public Health England (PHE) database. Participants were asked to self-complete a baseline questionnaire providing demographic data at enrolment (on paper), monthly sexual behaviour and adherence questionnaires (on paper or online), daily sexual behaviour and adherence diaries (on paper or online), and an acceptability questionnaire at the 12 month follow-up visit (on paper). The acceptability questionnaire was developed by the social science advisory group and is available as supporting information file S1 Questionnaire. Paper questionnaires were placed in an envelope, sealed and posted to the central data management team for data entry in MACRO database.

We used the Pearson chi-squared test, or the Fisher exact test where numbers were five or less in any group, to compare responses to the acceptability questions based on trial arm allocation (immediate or deferred), age group (34 and below or 35 and above, split at the median age of the cohort), educational level (degree or below), ethnicity (white/Irish or Black, Asian and minority ethnicity [BAME]), clinic of enrolment (inside or outside London), employment status (full/part-time employed or not) and place of birth (UK or not). Quantitative data were analysed using Stata 14.

In addition to categorical questions about acceptability, we asked participants to write what they most and least liked about the study and coded the free-text responses in Excel using emergent themes. The first author coded the free-text and two co-authors (EB, WN) checked the coding for consistency.

From February 2014 to January 2015, we purposely sampled 25 study participants to take part in semi-structured in-depth interviews (IDIs) which included a discussion topic on study acceptability. Participants were selected based on trial arm allocation (immediate or deferred), changes in their self-reported risk behaviour since enrolment (increased risk or same/decreased risk), and PrEP adherence among participants in the immediate group (high or low). We aimed to interview an equal number of participants in each of the six groups. During the interviews, we asked participants about the acceptability of the study design and procedures. Researchers who were independent of the study clinic team conducted the IDIs. Participants provided written informed consent and the IDIs were conducted in English and were audio-recorded. The recordings were transcribed, coded and analysed using framework analysis in NVivo 10 [30].

The PROUD study protocol was approved by London Bridge Research Ethics Committee, the Medicines and Healthcare products Regulatory Agency and each of the 12 participating Hospital Trusts (see list in acknowledgments). The trial is registered with ISRCTN (number ISRCTN94465371) and ClinicalTrials.gov (NCT02065986). The study protocol, including participant information sheet (PIS) and informed consent form (ICF), and the in-depth interview PIS, ICF and interview guide, are available on the study website.

## Results

In order to contextualise the acceptability of the study design, we present this in the context of the overall acceptability of the study. As such, we present the characteristics of the study cohort, acceptability of the study design, acceptability of the study visits, and acceptability of the study procedures. Within each section we present the quantitative data from the acceptability questionnaire, the free text responses from the acceptability questionnaire which are presented as proportions of responses not participants, and qualitative data from the in-depth interviews. To reduce the size of the tables, we make the data by employment status and date of birth available in supporting information file S1 Table as we observed no statistically significant differences in these analyses.

### Characteristics of the study cohort

#### Quantitative data

Of 544 (immediate [I] = 275, deferred [D] = 269) participants enrolled, 415 (76%) completed an acceptability questionnaire. One hundred and twenty nine participants (I = 50, D = 79) did not complete a questionnaire. Of these 21 (I = 5, D = 16) did not return to the PROUD study clinic after randomisation, 60 (I = 25, D = 35) did not have a visit at or after the scheduled 12 month visit, and 48 (I = 20, D = 28) had a visit at or after the scheduled 12 month visit but did not complete the questionnaire.

A higher proportion of participants in the immediate (82%) compared to deferred (71%) randomisation groups completed the acceptability questionnaire (p = 0.002) (Table 1). Participants who completed the acceptability questionnaire were older than participants who did not, at a median age of 36 (IQR 30–43) compared to 32 (IQR 28–40, p = 0.002). When controlling for age, participants in the immediate group were almost twice as likely to have completed the questionnaire than participants in the deferred group (AOR: 1.86, 95% CI 1.24, 2.81, p = 0.003). There was no evidence of differences between participants who completed the questionnaire based on education level, ethnicity, clinic of enrolment (Table 1), employment status or place of birth (available as supporting information file S1 Table).

**Table 1:** Comparison of participants who complete an acceptability questionnaire to those who didn't.

		Completed Questionnaire	
	All (col %)	Yes (row %)	P-value
	544 (100%)	415 (76%)	** **
**Trial arm**			**0.002**
Immediate	275 (51%)	225 (82%)	
Deferred	269 (49%)	190 (71%)	
**Age**			**0.002**
<25	54 (10%)	33 (61%)	
25–34	210 (39%)	152 (72%)	
35–44	178 (33%)	145 (81%)	
>45	98 (18%)	83 (85%)	
**Education**			0.178
Less than degree	213 (39%)	156 (73%)	
Degree	327 (61%)	256 (78%)	
**Employed**			0.369
No	100 (19%)	73 (73%)	
Yes	439 (81%)	339 (77%)	
**UK Born**			0.517
No	217 (40%)	169 (78%)	
Yes	322 (60%)	243 (75%)	
**Ethnicity**			0.524
BAME	100 (19%)	74 (74%)	
White/Irish	439 (81%)	338 (77%)	
**Clinic**			0.886
In London	381 (70%)	290 (76%)	
Out of London	163 (30%)	125 (77%)	

The median time from enrolment to completion of the acceptability questionnaire was 12.13 months (IQR 11.80, 12.80). Of 190 participants in the deferred group who completed the acceptability questionnaire, 125 completed it when first prescribed PrEP but before PrEP initiation, 45 after starting PrEP, 11 before starting, and seven completed it but never started PrEP (missing date of completion for two participants).

#### Free text data

Of the 415 (I = 225, D = 190) participants who completed the acceptability questionnaire, 369 (I = 204, D = 165) reported 589 (I = 323, D = 266) free text items that they most liked about the study. Forty-one percent (239/589) of the items reported related to the benefits of PrEP and HIV prevention more broadly, this equates to 45% (146/323) of all issues raised by the immediate group and 35% (93/266) of all issues raised by participants in the deferred group. Fifty-nine percent (350/589) of the items reported related to study acceptability. The key points raised about the acceptability of the study are presented in order of reporting in Table 2 and are discussed below. Prioritisation of issues hardly differed between participants in the immediate and deferred groups, and the narratives within the issues were very similar.

**Table 2:** What participants most liked about the study.

	All		Immediate		Deferred	
**Contributing to research**	110	31%	61	34%	49	28%
**Study staff**	97	28%	48	27%	49	28%
**HIV/STI testing**	71	20%	30	17%	41	24%
**Easy to participate**	20	6%	13	7%	7	4%
**Access to support/referrals**	21	6%	8	5%	13	8%
**Information**	18	5%	11	6%	7	4%
**Recording sexual behaviour**	11	3%	6	3%	5	3%
**All/no comment**	2	1%	0	0%	2	1%
**TOTAL**	350		177		173	

Of the 415 participants who completed the questionnaire, 315 (I = 179, D = 136) provided 360 (I = 199, D = 161) responses when asked what they least liked about the study. Fourteen percent (51/360) of the items reported related to the use of PrEP (I = 32, D = 19). Eighty six percent (309/360) of the items reported related to study acceptability and are presented in Table 3 and discussed below. In this analysis, 25% of participant responses reported there was nothing they disliked about the study, which was almost twice as high in the immediate group (32%) compared to the deferred group (17%).

**Table 3:** What participants least liked about the study.

	All		Immediate		Deferred	
**Nothing**	78	25%	54	32%	24	17%
**Data**						
Paper questionnaires	45	15%	26	16%	19	13%
diary	37	12%	21	13%	16	11%
data	27	9%	11	7%	16	11%
online	23	7%	15	9%	8	6%
**Study**						
visits	34	11%	18	11%	16	11%
deferred	27	9%	0	0%	27	19%
tests	22	7%	12	7%	10	7%
procedures	6	2%	3	2%	3	2%
feedback	5	2%	3	2%	2	1%
staff	2	1%	1	1%	1	1%
support	2	1%	2	1%	0	0%
recording	1	0%	1	1%	0	0%
TOTAL	309		167		142	

#### Qualitative data

We conducted 25 IDIs with 11 participants in the immediate and 14 in the deferred group. Categorisation based on the risk and adherence matrix used for purposeful sampling is shown in the figure in S1 Fig. The participants were enrolled across seven clinics, two of which were outside of London. Participants were a median age of 37 (range 25 to 55 years), 23 were in employment and 16 were educated to degree level. Twenty-four male (23 gay and one bisexual) and one trans female participated in IDIs. At the time of interview, participants had been enrolled in the study for a median of 12 months (range five to 20 months).

### Acceptability of study design

#### Quantitative data

Overall, 97% of participants reported being ‘glad they joined the study’, with only one person who was randomised to the immediate group disagreeing with this statement (Table 4). The vast majority (96%) expected to remain enrolled for the duration of the study. Eighty-seven percent were interested in joining another PrEP study after PROUD if available, however this differed by trial arm with 92% in the immediate group and 81% in the deferred group interested in joining another PrEP study (p = 0.002). Despite these high levels of study acceptability, 74% thought that the chance of being in the deferred group might put other volunteers off joining the study.

**Table 4:** Acceptability of the study design.

	Trial arm allocation				Age			Education			Ethnicity			Clinic		
	ALL	Immediate	Deferred	P-value	< = 34	> = 35	P-value	No Degree	Degree	P-value	BAME	White	P-value	London	Out of London	P-value
	** **	**225 (54%)**	**190 (46%)**	** **	**185 (45%)**	**228 (55%)**	** **	**156 (38%)**	**256 (62%)**	** **	**74(18%)**	**338 (82%)**	** **	**290 (70%)**	**125 (30%)**	** **
**Here are some statements that men might say about the PROUD study.**																
**I am glad I joined the PROUD study**				0.239			0.383			1.000			0.213			1.000
Agree	397 (97%)	216 (98%)	181 (96%)		177 (97%)	218 (96%)		151 (97%)	243 (96%)		69 (96%)	325 (97%)		276 (97%)	121 (98%)	
neutral/uncertain	12 (3%)	4 (2%)	8 (4%)		4 (2%)	8 (4%)		4 (3%)	8 (3%)		2 (3%)	10 (3%)		9 (3%)	3 (2%)	
Disagree	1 (<0.5%)	1 (<0.5%)	0 (0%)		1 (1%)	0 (0%)		0(0%)	1 (<0.5%)		1 (1%)	0		1 (<0.5%)	0	
**I expect to stay in the study for the whole 2 years**				0.365			1.000			0.921			1.000			0.904
Agree	392 (96%)	214 (97%)	178 (94%)		174 (96%)	216 (96%)		148 (96%)	241 (96%)		70 (97%)	319 (95%)		274 (96%)	118 (95%)	
neutral/uncertain	14 (3%)	5 (2%)	9 (5%)		6 (3%)	8 (3%)		5 (3%)	9 (3%)		2 (3%)	12 (4%)		9 (3%)	5 (4%)	
Disagree	4 (1%)	2 (1%)	2 (1%)		2 (1%)	2 (1%)		2 (1%)	2 (1%)		0	4 (1%)		3 (1%)	1 (1%)	
**I would like to join another PrEP study after this**				**0.002**			0.621			0.562			0.502			0.197
Agree	356 (87%)	203 (92%)	153 (81%)		155 (85%)	199 (88%)		131 (84%)	222 (88%)		60 (83.5%)	293 (87%)		243 (85%)	113 (91%)	
neutral/uncertain	47 (11%)	17 (8%)	30 (16%)		23 (13%)	24 (11%)		21 (14%)	26 (10%)		11 (15.5%)	36 (11%)		38 (13%)	9 (7%)	
Disagree	7 (2%)	1 (<0.5%)	6 (3%)		4 (2%)	3 (1%)		3 (2%)	4 (2%)		1 (1%)	6 (2%)		5 (2%)	2 (2%)	
**I think the chance of being in the deferred group and not getting Truvada for a year might put other men off joining the study**				0.798			0.234			0.624			0.507			0.352
Agree	301 (74%)	164 (75%)	137 (73%)		133 (73%)	166 (75%)		116 (76%)	183 (74%)		50 (71%)	248 (75%)		203 (72%)	98 (79%)	
neutral/uncertain	67 (17%)	36 (17%)	31 (17%)		35 (10%)	32 (14%)		26 (17%)	41 (16%)		11 (16%)	56 (17%)		50 (18%)	17 (14%)	
Disagree	37 (9%)	18 (8%)	19 (10%)		13 (7%)	24 (11%)		11 (7%)	25 (10%)		9 (13%)	28 (8%)		28 (10%)	9 (7%)	
**The written information I was given clearly explained the study**				0.313			0.184			**0.021**			0.480			0.811
Agree	399 (98%)	216 (98%)	183 (97%)		178 (98%)	219 (97%)		155 (100%)	241 (96%)		71 (100%)	325 (97%)		277 (97%)	122 (98%)	
neutral/uncertain	9 (2%)	3 (1.5%)	6 (3%)		2 (1%)	7 (3%)		0	9 (4%)		0	9 (3%)		7 (2%)	2 (2%)	
Disagree	1 (<0.5%)	1 (<0.5%)	0 (0%)		1 (1%)	0 (0%)		0	1 (<0.5%)		0	1 (<0.5%)		1 (<0.5%)	0 (0%)	

The majority agreed that the participant information sheet clearly explained the study (98%), although this differed by educational level with 10 participants educated to degree level stating the information sheet did not clearly explain the study or being neutral on this point, compared to none with less than degree level education (p = 0.021). There are no additional data to indicate why they did not think the information sheet was clear. There were no other significant differences based on other demographic factors.

#### Free text data

In the free text data, 31% (110/350) of participant responses noted that being able to contribute to HIV prevention research was what they most liked about participating in the study, as this quote illustrates:

“It feels like I'm helping the gay community to fight and eradicate HIV” (deferred).

While no one in the immediate group cited the wait-listed design when asked what they liked least about the study, 20% (27/136) of participants in the deferred group noted ‘being deferred’ as the thing they liked least. This was the second most common reported item disliked after the data collection tools, even though it only accounted for 19% (27/142) of all least liked issues raised by deferred participants:

“I was slightly disappointed to be put in the deferred treatment group but understand the necessity of this” (deferred).

“Having to wait a year to start PrEP during the highest risk period of my life” (deferred).

#### Qualitative data

Participants in the IDIs had a good understanding of the rationale for the open-label wait-listed study design in terms of assessing effectiveness, adherence, changes in risk behaviour, acquisition of other STIs, and the cost-effectiveness of an NHS funded PrEP programme.

Seventeen participants (I = 6, D = 11) discussed the acceptability of the wait-listed study design in some detail when asked about it. Most accepted the design with participants in the immediate group stating they would have been disappointed to be randomised to the deferred group but would have accepted it. Similarly, participants in the deferred group stated they were disappointed to be randomised to the deferred group but accepted it. The general sense was that volunteers knew what they were signing up to:

“I was slightly disappointed because I knew I was at risk by my behaviour at the time, I was hoping to get on it (PrEP) for the full 2 years, but obviously it was always made clear right from the start that there was a deferred group and there was an immediate group, so I couldnt complain. It soon came around anyway and it was in time, so (laughter)” (deferred participant who started PrEP before the IDI).

The issue raised in this quote about hoping to access PrEP ‘in time’ before contracting HIV emerged in a hand-full of interviews with deferred participants:

“If I get infected in the first year I would be pretty angry I think…. we know that this exists and we don’t (have it), so why not… I can almost predict that I will run into something (test positive) at the last test…. then I will be quite upset”.

A participant who sero-converted during the deferred period described his experience as follows:

“Knowing how effective Truvada is I think everyone would rather be in the immediate rather than the control, because obviously your chances are better, but it is a 50/50 chance a roll of the dice, I wasn’t particularly put off or anything by being in the deferred”.

Only one participant, who had used PrEP before joining the study, said he had not understood there was a chance of being deferred until he was deferred, but accepted the randomisation:

“The 12 months actually gave me a chance to think about it (PrEP) again, rather than just taking a tablet, but actually do I need it, do I want it. So actually I reassessed my situation, whether I should use PrEP or not use PrEP and how I sexually behave whilst using PrEP or not using PrEP”.

Only five (I = 1, D = 4) participants specifically objected to the wait-listed design. A participant in the immediate group said he probably would not have returned to the clinic if he had been randomised to the deferred group. A participant in the deferred group described how he had planned to accept being randomised to either group, but after spending many hours in the clinic due to delays, was “incandescent with rage” when finally randomised to the deferred group. Instead of returning to the clinic, he enrolled a second time at a different clinic and was randomised to the immediate group (he was one of two co-enrolments excluded from the final analyses). The other three deferred participants were not convinced of the need for the deferred group describing it as a ‘wasted year’ and ‘unfair’. In addition, two other participants interviewed, reported use of PrEP accessed outside of the trial during their deferred period in the trial.

### Acceptability of study visits

#### Quantitative data

The majority of participants didn’t mind visiting the clinics quarterly (89%), liked having regular HIV (93%) and STI (87%) screening, and felt there were able to access as much support as they needed to reduce the risk of HIV and other STIs (90%) (Table 5). However, 8% of older participants compared to only 4% of younger participants did not like regular STI screening (p = 0.027). Although a similar proportion of BAME and white participants felt that they were able to access as much support as they needed to reduce their risk behaviour (92% v 90%), there were differences in their reporting of a neutral (4% v 10%) or negative response (4% v 1%), (p = 0.029). However, these differences are difficult to interpret with such small numbers and differential representation of BAME participants across clinics.

**Table 5:** Acceptability of the study visits.

	Trial arm allocation				Age			Education			Ethnicity			Clinic		
	ALL	Immediate	Deferred	P-value	< = 34	> = 35	P-value	No Degree	Degree	P-value	BAME	White	P-value	London	Out of London	P-value
	** **	**225 (54%)**	**190 (46%)**	** **	**185 (45%)**	**228 (55%)**	** **	**156 (38%)**	**256 (62%)**	** **	**74(18%)**	**338 (82%)**	** **	**290 (70%)**	**125 (30%)**	** **
**Here are some statements that men might say about the PROUD study.**																
**Visiting the clinic every 3 months is not a problem**				0.182			0.931			0.858			0.173			0.918
Agree	366 (90%)	202 (92%)	164 (87%)		161 (89%)	203 (90%)		139 (90%)	224 (89%)		68 (94%)	295 (88%)		256	110 (89%)	
neutral/uncertain	33 (8%)	15 (7%)	18 (9%)		15 (8%)	18 (8%)		11 (7%)	22 (9%)		2 (3%)	31 (9%)		22	11 (9%)	
Disagree	10 (2%)	3 (2%)	7 (4%)		5 (3%)	5 (2%)		4 (3%)	6 (2%)		2 (3%)	8 (2%)		7	3 (2%)	
**I like having regular HIV tests**				0.889			0.790			0.471			0.121			1.000
Agree	380 (93%)	205 (94%)	175 (93%)		167 (92%)	211 (94%)		146 (95%)	231 (92%)		71 (99%)	306 (92%)		265 (93%)	115 (93%)	
neutral/uncertain	23 (6%)	12 (5%)	11 (6%)		11 (6%)	12 (5%)		6 (4%)	17 (7%)		1 (1%)	22 (7%)		16 (6%)	7 (6%)	
Disagree	5 (1%)	2 (1%)	3 (1%)		3 (2%)	2 (1%)		2 (1%)	3 (1%)		0 (0%)	5 (1%)		4 (1%)	1 (1%)	
**I do not like having regular STI tests**				0.910			**0.027**			0.949			1.000			0.331
Agree	26 (6%)	13 (6%)	13 (7%)		7 (4%)	18 (8%)		10 (6.5%)	15 (6%)		4 (6%)	21 (6%)		19 (7%)	7 (6%)	
neutral/uncertain	28 (7%)	15 (7%)	13 (7%)		18 (19%)	10 (4%)		10 (6.5%)	18 (7%)		5 (7%)	23 (7%)		23 (8%)	5 (4%)	
Disagree	355 (87%)	193 (87%)	162 (86%)		156 (86%)	198 (88%)		134 (87%)	219 (87%)		62 (87%)	291 (87%)		244 (85%)	111 (90%)	
**I am able to access as much support to reduce my risk of HIV and STIs as I need**				0.786			0.713			0.295			**0.029**			0.360
Agree	368 (90%)	201 (91%)	167 (89%)		161 (89%)	205 (91%)		142 (93%)	223 (88%)		65 (92%)	300 (90%)		253 (89%)	115 (93%)	
neutral/uncertain	35 (9%)	18 (8%)	17 (9%)		17 (9%)	18 (8%)		9 (6%)	26 (10%)		3 (4%)	32 (9%)		26 (9%)	9 (7%)	
Disagree	5 (1%)	2 (1%)	3 (2%)		3 (2%)	2 (1%)		2 (1%)	3 (1%)		3 (4%)	2 (1%)		5 (2%)	0 (0%)	
**Some men have been, or will be, asked to give additional blood samples so as laboratory tests can measure the level of Truvada in their blood. These tests can tell how regularly a**																
**I think it is a good idea to check how regularly men are taking their tablets by measuring the level of Truvada in their blood**				1.000			0.586			0.501			0.230			0.789
Agree	392 (96%)	211 (96%)	181 (96%)		176 (97%)	214 (95%)		147 (95%)	242 (96%)		67 (93%)	322 (96%)		274 (96%)	118 (96%)	
neutral/uncertain	15 (4%)	8 (4%)	7 (4%)		6 (3%)	9 (4%)		7 (5%)	8 (3%)		5 (7%)	10 (3%)		11 (4%)	4 (3%)	
Disagree	2 (<0.5%)	1 (<0.5%)	1 (<0.5%)		0 (0%)	2 (1%)		0 (0%)	2 (1%)		0 (0%)	2 (1%)		1 (<0.5%)	1 (1%)	
**Men report how regularly they take their tablets so there is no need to measure the level of Truvada in their blood**				0.300			0.397			0.951			0.124			0.735
Agree	65 (16%)	31 (14%)	34 (18%)		24 (13%)	40 (18%)		25 (16%)	38 (15%)		17 (24%)	47 (14%)		43 (15%)	22 (18%)	
neutral/uncertain	120 (29%)	61 (28%)	59 (31%)		57 (31%)	62 (27%)		45 (29%)	74 (29%)		18 (25%)	101 (30%)		86 (30%)	34 (28%)	
Disagree	224 (55%)	128 (58%)	96 (51%)		101 (56%)	123 (55%)		84 (55%)	140 (56%)		37 (51%)	186 (56%)		157 (55%)	67 (54%)	
**When I am on Truvada, I would like to find out the level of Truvada in my blood**				0.771			0.863			0.576			0.683			0.637
Agree	350 (86%)	186 (84%)	164 (87%)		157 (86%)	191 (85%)		134 (87%)	213 (85%)		64 (89%)	283 (85%)		242 (85%)	108 (88%)	
neutral/uncertain	53 (13%)	30 (14%)	23 (12%)		23 (13%)	30 (13%)		19 (12%)	34 (13%)		7 (10%)	46 (14%)		40 (14%)	13 (10%)	
Disagree	6 (1%)	4 (2%)	2 (1%)		2 (1%)	4 (2%)		1 (1%)	5 (2%)		1 (1%)	5 (1%)		4 (1%)	2 (2%)	

Pharmacokinetic samples were collected from 59 participants, but all participants were asked about the hypothetical acceptability of drug level monitoring to assess adherence in a future trial. In a series of three questions, 96% of participants thought that drug level monitoring was a good idea, 86% reported they would like to know their drug levels when on PrEP, and only 16% felt that there was no need to conduct drug level testing to assess adherence. These perspectives did not differ by trial allocation or baseline demographics.

#### Free text data

In the free text data, a fifth (20%) of participant responses related to the benefits of regular HIV and STI testing in terms of helping them manage their sexual health by providing a structure for regular testing. Twenty-eight percent of participant responses reported the service they received from the study staff as the aspect of the study they most liked. The following descriptors were used in relation to the staff (in alphabetical order):

‘Accessible, amazing, approachable, caring, courteous, efficient, friendly, great, helpful, honest, having integrity, incredible, informative, kind, non-judgement, openly talk about sex, patient, pleasant, polite, professional, reassuring, supportive, trustworthy and understanding’.

Participants particularly appreciated the consistency of care by being able to see the same health professionals regularly and build a relationship with the clinical team, as shown in this response:

I most liked.. “the offer of seeing one designated person every 3 months for a check-up rather than being passed from person to person in a clinic” (deferred)

Other points that participants most liked included the ease of attending study visits and adhering to study procedures (6%), access to support and referral systems (6%), access to additional information about the study, study progress and HIV prevention more generally (5%).

Least liked aspects of the study visits included difficulties of getting appointments and travel time to the clinics (11%), having blood taken and fear of needles (7%), specific procedures (recruitment, enrolment, aims unclear, partner enrolment, recruitment closing) (2%), lack of feedback in terms of an online forum or support phone line (2%), poor treatment by staff (1%), and lack of support (1%).

#### Qualitative data

The qualitative data reinforces findings from the quantitative data with the majority of participants reporting that they liked the regular clinic visits and HIV and STI screening. However, three participants did comment on the “minor inconvenience” of quarterly visits, and the difficulty of attending clinics especially during working hours. A few participants would have preferred pre-booked quarterly appointments with visit reminders. Another participant liked the convenience of being able to go to any clinic, not just the study clinic, for HIV and STI screening. A participant in the deferred group had not attended the clinic for six months and felt that, without the motivation of PrEP to attend, it was easy to lose touch with the clinic. He thought that additional email contact with participants during the deferred period would ensure better retention.

As in the free text findings, participants in the IDIs praised the clinic staff without being prompted to talk about them, especially the fact that they were welcoming, respectful and non-judgemental. They also highlighted the benefit of seeing the same staff members regularly in the clinic, as this quote highlights:

“It is really important to me… I don’t know him very well, but I have been able to disclose a few things to him… It means it is not caught up in my head, I am not denying it, I am not avoiding it, I am not parking it in the ether, and I wouldn’t be able to do that if it was a random team member …even though I don’t know him very well the relational aspect is really important”.

### Acceptability of study data collection tools

#### Quantitative data

Acceptability of the data collection tools differed by individual tool with 82% of participants reporting they didn’t mind completing the monthly sexual behaviour questionnaires, 56% reporting they didn’t mind completing the daily sexual behaviour and adherence diary, and 56% reporting liking completing the questionnaires online (Table 6). However, there was evidence of a difference by trial arm in the proportion of participants who agreed (22% in immediate, 16% in deferred) or were neutral or uncertain (20% in immediate, 30% in deferred) in response to the statement that they disliked completing the daily diary (p = 0.036).

**Table 6:** Acceptability of the study data collection.

	Trial arm allocation				Age			Education			Ethnicity			Clinic		
	ALL	Immediate	Deferred	P-value	< = 34	> = 35	P-value	No Degree	Degree	P-value	BAME	White	P-value	London	Out of London	P-value
	** **	**225 (54%)**	**190 (46%)**	** **	**185 (45%)**	**228 (55%)**	** **	**156 (38%)**	**256 (62%)**	** **	**74(18%)**	**338 (82%)**	** **	**290 (70%)**	**125 (30%)**	** **
**Here are some statements that men might say about the PROUD study.**																
**I don’t mind completing the monthly sexual behaviour questionnaires**				0.282			0.284			0.687			0.251			0.152
Agree	337 (83%)	188 (85%)	149 (79%)		143 (79%)	192 (85%)		128 (83%)	206 (82%)		54 (76%)	280 (84%)		228 (80%)	109 (88%)	
neutral/uncertain	54 (13%)	24 (11%)	30 (16%)		28 (15%)	26 (11%)		21 (14%)	33 (13%)		12 (17%)	42 (12%)		43 (15%)	11 (9%)	
Disagree	18 (4%)	9 (4%)	9 (5%)		10 (6%)	8 (4%)		5 (3%)	13 (5%)		5 (7%)	13 (4%)		14 (5%)	4 (3%)	
**I dislike completing the sexual behaviour diary**				**0.036**			**0.019**			0.925			0.975			0.189
Agree	78 (19%)	49 (22%)	29 (16%)		41 (23%)	36 (16%)		30 (20%)	46 (19%)		14 (20%)	63 (19%)		55 (19%)	23 (19%)	
neutral/uncertain	102 (25%)	45 (21%)	57 (30%)		52 (29%)	49 (22%)		37 (24%)	64 (25%)		17 (24%)	83 (25%)		78 (28%)	24 (19%)	
Disagree	227 (56%)	126 (57%)	101 (54%)		87 (48%)	140 (62%)		85 (56%)	142 (56%)		39 (56%)	188 (56%)		151 (53%)	76 (62%)	
**I like completing the questionnaires on-line**				0.706			**0.007**			0.158			0.091			**0.014**
Agree	224 (56%)	124 (57%)	100 (54%)		85 (48%)	138 (62%)		83 (55%)	139 (56%)		32 (46%)	191 (58%)		146 (52%)	78 (65%)	
neutral/uncertain	125 (31%)	64 (29%)	61 (33%)		69 (39%)	55 (25%)		42 (28%)	82 (33%)		24 (34%)	99 (30%)		100 (35%)	25 (21%)	
Disagree	53 (13%)	30 (14%)	23 (13%)		24 (13%)	29 (13%)		26 (17%)	27 (11%)		14 (20%)	39 (12%)		36 (13%)	17 (14%)	
**I would prefer to answer the sexual behaviour questions on a mobile device**				0.891			**<0.001**			0.459			0.821			0.291
Agree	238 (58%)	126 (57%)	112 (59%)		132 (73%)	105 (46%)		90 (58%)	146 (58%)		44 (61%)	192 (57%)		160 (56%)	78 (63%)	
neutral/uncertain	126 (31%)	70 (31%)	56 (30%)		42 (23%)	83 (37%)		44 (28%)	81 (32%)		20 (28%)	105 (31.5%)		90 (31%)	36 (29%)	
Disagree	46 (11%)	25 (11%)	21 (11%)		8 (4%)	38 (17%)		21 (14%)	25 (10%)		8 (11%)	38 (11.5%)		36 (13%)	10 (8%)	
**Accurately answering questions about sexual behaviour can be difficult as some people forget details or don’t want to share all details.**																
**When completing the questionnaires, I am able to report my sexual activity honestly**				0.347			0.180			0.298			0.607			0.480
Agree	354 (88%)	196 (90%)	158 (85%)		151 (85%)	202 (91%)		128 (85%)	224 (90%)		62 (86%)	291 (89%)		250 (87%)	104 (90%)	
neutral/uncertain	39 (10%)	17 (8%)	22 (12%)		22 (12%)	16 (7%)		18 (12%)	20 (8%)		9 (13%)	29 (9%)		28 (10%)	11 (9%)	
Disagree	10 (2%)	5 (2%)	5 (3%)		5 (3%)	5 (2%)		5 (3%)	5 (2%)		1 (1%)	8 (2%)		9 (3%)	1 (1%)	
**When completing the questionnaires, I find it difficult to remember my sexual activity in the last 30 days**				0.441			**0.026**			0.935			0.815			0.558
Agree	188 (47%)	105 (48%)	83 (45%)		96 (54%)	92 (41%)		70 (46%)	117 (47%)		32 (44.5%)	155 (47%)		138 (48%)	50 (43%)	
neutral/uncertain	69 (17%)	40 (18%)	29 (16%)		23 (13%)	45 (20%)		27 (18%)	41 (16%)		14 (19.5%)	54 (17%)		46 (16%)	23 (20%)	
Disagree	146 (36%)	73 (33%)	73 (39%)		59 (33%)	86 (39%)		54 (36%)	91 (37%)		26 (36%)	119 (36%)		103 (36%)	43 (37%)	
**I find the questionnaires make it difficult to be accurate about my sexual activity**				0.668			0.550			0.638			0.280			0.076
Agree	113 (28%)	65 (30%)	48 (26%)		55 (31%0	58 (26%)		45 (30%)	68 (27%)		24 (33%)	89 (27%)		72 (25%)	41 (35%)	
neutral/uncertain	110 (27%)	57 (26%)	53 (29%)		47 (26%)	62 (28%)		43 (28%)	65 (26%)		22 (31%)	87 (27%)		85 (30%)	25 (22%)	
Disagree	180 (45%)	96 (44%)	84 (45%)		76 (43%)	103 (46%)		63 (42%)	116 (47%)		26 (36%)	152 (46%)		130 (45%)	50 (42%)	

Younger participants disliked the sexual behaviour diary more than older participants (23% v 16%, p = 0.019) and older participants liked completing the questionnaires online more than younger participants (62% v 48%, p = 0.007). Similarly, participants enrolled outside of London liked completing the questionnaires online more than participants enrolled in London (65% v 52%, p = 0.014).

Fifty-eight percent of participants reported they would have preferred to answer the sexual behaviour questions on a mobile device (i.e. smart phone or tablet). This was in comparison to being able to answer the questions on a paper questionnaire or via the on-line database. As above, this differed by age with 73% of younger compared to only 47% of older participants being interested in this option (p<0.001).

In terms of self-completed questionnaires, 88% felt they could report sexual activity honestly, although 47% found it difficult to remember sexual activity over the last 30 days and 28% thought the questionnaires made it difficult to accurately report sexual activity. This differed by age with 54% of younger compared to 41% of older participants finding it difficult to remember their sexual behaviour (p = 0.026).

#### Free text data

In the free text responses, the data collection tools were the most frequently reported least liked aspect of the study by participants in both groups. A total of 132 participants (I = 73, D = 59) mentioned at least one data related item that they disliked: 15% of responses related to the monthly self-completed questionnaires, 12% to the daily diary, 9% to data collection generally, and 7% specifically about the online participant website where participants could complete the questionnaires and diary.

Participants said that there were too many forms to complete with the monthly sexual behaviour and adherence questionnaire and daily diary and that it was difficult to remember to complete them. They described the questionnaires and diary as being cumbersome, fiddly, a chore to complete and time consuming. In addition, the questionnaires were described as being too long, frustrating, repetitive and unclear, and the diary as being awkward, laborious, not user friendly, a pain, and tedious, as this quote demonstrates:

“The online diary site is *SUCH* a pain! Having to click into each day separately is very laborious” (deferred, emphasis by participant).

Participant suggestions included having email reminders to complete the questionnaire and diary, to have longer than the 2 week cut off point to complete the last months questionnaire, to be able to view the diary in order to complete the monthly questionnaire, and to be able to block edit days in the diary, repeat data from the previous day or merely complete the diary when you had missed a tablet or had sex.

A number of participants complained that it was difficult to recall sexual behaviour when completing the questionnaire and diary. A few also complained that they were not able to report their behaviour as accurately as they would have liked as the questionnaire and diary did not allow them to report behaviour that was not requested (i.e. group sex, ejaculation), or to elaborate on reasons for specific behaviours, for example:

“Diary doesn't cover all issues, what about non anal sex partners? More than 1 partner a day? Other reasons for not taking pills other than forgot e.g. illness or travel” (immediate)

A number of participants described the online website as difficult to access, unreliable and ‘clunky’, with some arguing that they would have preferred a mobile application. One person raised a concern about data protection issues with the online website.

While 1% of least liked responses related to recalling numbers of sexual partners, 3% of most liked responses related to participants liking the need to recall and record their sexual behaviour as it made them review their behaviour and consider changes:

“It made me feel more aware of my sexual health and has made me less likely to be unsafe than one year ago” (immediate).

#### Qualitative data

In the qualitative interviews, there was almost an even split between people who liked and didn’t like completing the questionnaires and diaries. Much of the negative feedback received in the free text responses was repeated in the IDIs including the fact that the data collection tools were duplicative, questions were ambiguous, and the diaries were difficult to complete especially in terms of having to enter data about not having sex and not taking PrEP each day. In addition, participants reported that they rarely completed the diary daily, more likely weekly or monthly either from memory or from personal diaries or even spreadsheets.

As in the free text responses, some participants were frustrated that the questionnaire and diary were restrictive, not allowing participants to add comments or explain entries. In the IDIs, it was clear that this mainly related to participants wanting an opportunity to explain their rationale for risk taking or actions that may be perceived by the categorical approach of data collection to be more risky than the participant felt they actually were.

Out of 12 participants who talked about the accuracy of their self-reported data, half thought they were very accurate and half didn’t, mainly due to forgetting to complete the diary regularly, difficulty recalling their behaviour accurately, and difficulty being as accurate as they wanted to be given the categorical nature of the data collection tools.

Recommendations on how to improve data collection included reminders for both visits and data completion, completing the data on a mobile application, having questionnaires administered instead of self-completed which one participant did not think would increase social desirability bias, and more opportunities for participants to describe their behaviour instead of ticking categorical options.

## Discussion

This is the first study to assess the acceptability of using an open-label wait-listed design to evaluate the effectiveness of a new biomedical HIV prevention technology. The first concern when designing the trial was the possibility that randomisation to the deferred group would discourage volunteers from enrolling. Although we enrolled to target, accrual was slower than expected, partly due to limited PrEP awareness [28]. In this paper, we show that three-quarters of participants thought the wait-listed design would discourage other volunteers from joining a similar study. This evidence suggests that the wait-listed design probably contributed to the slower than anticipated rate of recruitment. This is an important consideration in planning future wait-listed trials.

The second concern when designing the trial was that retention would be lower in the deferred-group, potentially compromising the validity of the trial result. We reported previously that participants in the immediate-group attended clinic more regularly than deferred-group participants to collect their PrEP prescriptions, with a mean of 4.2 compared to 3.6 visits in the first year [29]. Without the facility to identify new HIV sero-conversions via the PHE database, this could have affected the outcome of the trial. Indeed, a fifth of deferred participants reported the wait-listed design as an element they disliked about the study. Given completion of the acceptability questionnaire was significantly lower among participants in the deferred than the immediate group, this is likely to underestimate the extent to which participants disliked the wait-listed design. However, it is interesting to note that in free-text data, deferred participants disliked the data collection tools more than the fact that they had to wait a year to access PrEP. In fact, overall, participants had more reservations about the data collection tools than any other aspect of the study design, visits or procedures. The quantitative and qualitative findings suggest that in an environment where PrEP was not available, the rationale for the wait-listed design was well understood and generally acceptable to most participants.

Our findings on the acceptability of study visits provide some lessons for PrEP implementation, as well as future prevention research. In England, MSM are advised to have HIV and STI screening at least annually, and every 3 months if having condomless sex with new or casual partners [31]. At enrolment, participants reported screening for STIs a median of 3 (IQR 2–4) times in the previous 12-months [28] During the study, PROUD participants clearly found that in the context of PrEP provision, quarterly clinic visits and the routine of regular HIV and STI screening were acceptable. These findings suggest that among a cohort of MSM who are engaged with sexual-health services and perceive themselves to be at risk of HIV, regular clinic visits are likely to be acceptable in future PrEP programmes. However, we still know very little about the optimal ways to deliver PrEP to individuals at risk of HIV who are less engaged with sexual-health services or do not perceive their risk to be sufficient to access PrEP. Additionally, in a sub-sample of the study population we used drug level testing to confirm participant’s adherence to daily dosing. Among all participants, the idea of drug level testing was perceived as an acceptable procedure. While this finding is useful for future clinical trials, this type of adherence assessment is unlikely to be deemed necessary in a clinical setting in the UK.

Another lesson for implementation is the importance of seeing the same health professionals regularly in terms of building rapport with the staff. While participants felt they could access as much support as they needed to reduce their risk of HIV and STIs, we did not measure the type of support they wanted in the context of PrEP use. In the baseline cohort, 9.5% of participants reported moderate to severe depression and 44% reported the use of recreational drugs commonly associated with chemsex [28, 32]. In planning a PrEP implementation programme, we need to explore the types of support services that PrEP users require and the best ways to offer these services.

PROUD was originally designed as a pilot study and as such, feedback on the shortcomings of the data collection tools was valuable in preparation for a larger-scale RCT. We asked participants to self-complete sexual-behaviour and adherence questionnaires to reduce the impact of social desirability bias and reduce the clinic workload. Four-fifths of participants didn’t mind completing the questionnaires and over half didn’t mind completing the daily-diary. This is despite feedback that there were too many questionnaires, questions were often unclear, and the online data entry site was difficult to use. This suggests that while participants were critical of the data collection tools, the majority did not object to providing self-completed data and felt they were able to do so honestly. Of note, almost half of participants found it difficult to recall their sexual activity in the last 30 days. The key lessons are that the data collection tools need to be streamlined, for example by using clearer categorizations, cover shorter time-periods or be supported with aides-memoire, and available on multiple devices, including mobile apps, to meet a variety of individual preferences.

Participants were critical about the design of the questionnaires with over a quarter stating they made it difficult to accurately report sexual behaviour, an issue echoed in the free-text responses. While there is a lot of room for improvement of the questionnaires, this issue also relates to the challenge of collecting data on sexual risk behaviour in the current HIV landscape. We know from previous analysis that for participants in this study, it was clear that a ‘risk’ depended not only on condom use, but also PrEP use, a partners HIV-status and viral-load, whether the participant was top or bottom, and whether ejaculate was involved [33]. In the era of PrEP and treatment as prevention (TasP), we need to reconsider the questions we use to measure risk behaviour, taking account of both the need to streamline the data collection tools and realistically capture ‘risk’ from the perspective of participants. As noted by other authors, we need to adapt measures of risk to take account of the expanded HIV prevention options which include the use of ARVs by both positive and negative individuals, and move away from simplified notions of condomless sex being ‘unprotected’, ‘unsafe’ or a ‘risk’ factor for HIV acquisition [34, 35].

Although we were specifically interested in assessing acceptability of the trial design, it was useful to contextualise participant’s perceptions within the broader context of acceptability of study procedures more generally. This analysis benefits from that fact that we assessed acceptability of the study across participants in the immediate and deferred groups. However, one limitation is that a quarter of participants did not complete the acceptability questionnaire and they were almost twice as likely to be in the deferred group. We attempted to arrange in-depth interviews with participants who had stopped attending the clinic or withdrawn from the study, but were either unable to contact them or they were unwilling to attend an in-depth interview. Another limitation is that we relied on the self-completed sexual behaviour data to purposely select participants for the in-depth interviews, and therefore did not sample participants who did not complete any questionnaires. A further limitation is that although trans women were eligible to join the study, our recruitment strategy was not sufficiently trans inclusive and therefore only three trans women joined the study. As such, these findings only really reflect the views of the cis men who joined the study. Our findings highlight the benefit of using mixed method research approaches to assess the acceptability of study designs, procedures and data collection processes in preparation for large-scale clinical trials.

Our findings demonstrate that for PROUD, the open-label wait-listed RCT was an acceptable and feasible trial design. It was the optimal design to estimate the net-effect of PrEP in terms of biological efficacy, product adherence and risk compensation. It also enabled a comparison of behaviour change and rates of STIs between the groups, as well as HIV incidence. Without the deferred control-group, the study results would have been uninterpretable as HIV incidence in the intervention group was comparable to the expected HIV incidence among MSM attending sexual health clinics and yet incidence in the control-group was seven-times higher than expected [29]. The open-label wait-listed RCT design is not a panacea to the challenges that some placebo-controlled biomedical prevention trials have faced in terms of achieving sufficient adherence. However, in this case, an open-label wait-listed RCT was the optimal design to address the research question of interest in PROUD and provided the most valid measure of public health benefit.

Given the challenges of achieving sufficient adherence in recent biomedical HIV prevention trials, evidence from open-label wait-listed RCTs should be considered to support public health interventions, as in PROUD, as well as in support of licensing applications of new drugs where appropriate. The World Health Organisation now recommends PrEP for populations with an HIV incidence of three per 100 person-years, although access remains extremely limited in most countries [36]. Future biomedical HIV prevention trials will need to consider including oral PrEP as either the control group or an additional standard of care. While there may still be circumstances whereby the open-label wait-listed RCT remains ethically acceptable for the evaluation of new biomedical HIV prevention technologies, this trial design should be considered more broadly when the presence of a placebo could impede adherence sufficiently to undermine the power of an RCT.

## Supporting information

S1 Fig Purposive selection of participants for in-depth interviews based on trial arm allocation, risk behaviour and adherence. (PDF)

S1 Questionnaire Acceptability Questionnaire. (PDF)

S1 Table Employment and place of birth data tables. (DOCX)
