# Supplementary material for: Acceptability of an open-label wait-listed trial design: Experiences from the PROUD PrEP study
Source: PLoS One. 2017 Apr 20;12(4):e0175596. doi: 10.1371/journal.pone.0175596 (PMC5398545; doi:10.1371/journal.pone.0175596)
Supplement: S1 Fig — (PDF) [file pone.0175596.s001.pdf]

S1 Figure: Purposive selection of participants for in-depth interviews based on trial arm allocation, risk behaviour and adherence

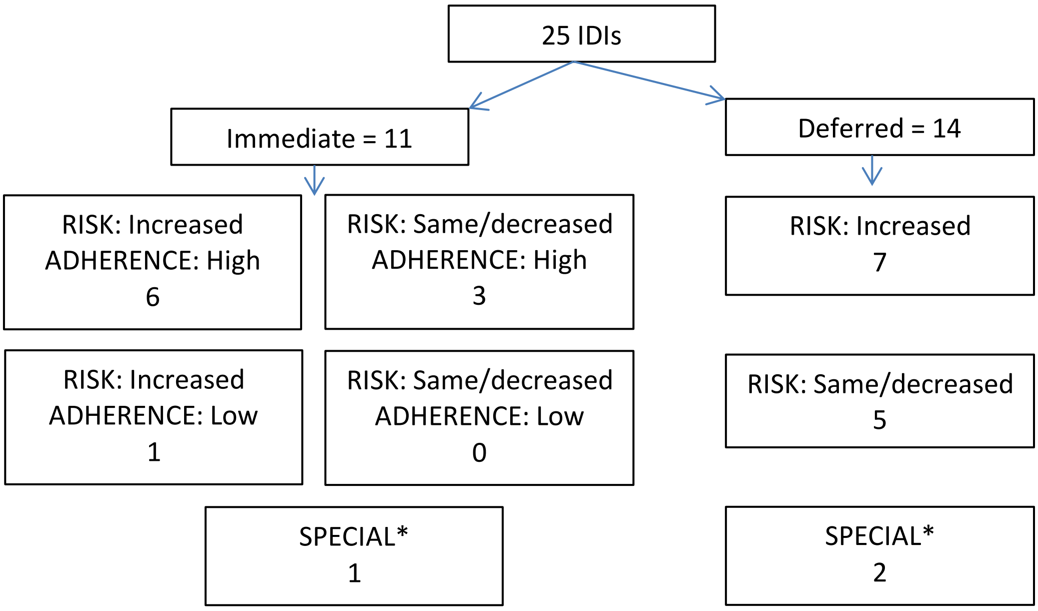

**Special interviews were conducted with a trans woman, a person who sero-converted during the study, and a person who decided not to start PrEP.*
