# Supplementary material for: Acceptability of an open-label wait-listed trial design: Experiences from the PROUD PrEP study
Source: PLoS One. 2017 Apr 20;12(4):e0175596. doi: 10.1371/journal.pone.0175596 (PMC5398545; doi:10.1371/journal.pone.0175596)
Supplement: S1 Questionnaire — (PDF) [file pone.0175596.s002.pdf]

Study Acceptability Questionnaire

Trial no:	Initials:	Date of birth:	Date form completed:
P ☐☐☐☐☐☐	☐☐☐	☐☐/☐☐/☐☐	☐☐/☐☐/☐☐

1. To the best of your memory, where did you first hear about the PROUD study?
Please tick one option:

☐ At a sexual health clinic
☐ From a sexual partner
☐ From a gay or transgender friend
☐ In the news or on the internet
☐ From another research project
☐ Other (please specify) _________________________

2. How much did each of the following influence your decision to join the PROUD study?
Please tick one box per row:

	Influenced very much	Influenced a little	Did not influence
Steady sexual partner(s)	☐	☐	☐
Non-steady sexual partner(s)	☐	☐	☐
Gay/transgender friend(s)	☐	☐	☐
Straight friend(s)	☐	☐	☐
Family member(s)	☐	☐	☐
News media about PrEP as HIV prevention	☐	☐	☐
PROUD study staff	☐	☐	☐
Clinical staff not in the PROUD study team	☐	☐	☐

3. Since joining the study, how much have you talked to each of the following about taking part in the PROUD study?
Please tick one box per row:

	Talked about a lot	Talked about a little	Have not talked about
Steady sexual partner(s)	☐	☐	☐
Non-steady sexual partner(s)	☐	☐	☐
Gay/transgender friend(s)	☐	☐	☐
Straight friend(s)	☐	☐	☐
Family member(s)	☐	☐	☐
Clinic staff not in the PROUD study team	☐	☐	☐

4. Here are some statements that men might say about the PROUD study.
Please tick one box per row to show how much you agree or disagree with each of these statements:

	Strongly Agree	Agree	Neutral or uncertain	Disagree	Strongly Disagree
I am glad I joined the PROUD study	☐	☐	☐	☐	☐
The written information I was given clearly explained the study	☐	☐	☐	☐	☐
I don't mind completing the monthly sexual behaviour questionnaires	☐	☐	☐	☐	☐
I dislike completing the sexual behaviour diary	☐	☐	☐	☐	☐
I like completing the questionnaires on-line	☐	☐	☐	☐	☐
Visiting the clinic every 3 months is not a problem	☐	☐	☐	☐	☐
I like having regular HIV tests	☐	☐	☐	☐	☐
I do not like having regular STI tests	☐	☐	☐	☐	☐
I am able to access as much support to reduce my risk of HIV and STIs as I need	☐	☐	☐	☐	☐
I think the chance of being in the deferred group and not getting Truvada for a year might put other men off joining the study	☐	☐	☐	☐	☐
I expect to stay in the study for the whole 2 years	☐	☐	☐	☐	☐
I would like to join another PrEP study after this	☐	☐	☐	☐	☐
I would prefer to answer the sexual behaviour questions on a mobile device (i.e. smart phone or tablet)	☐	☐	☐	☐	☐

5. Some men have been, or will be, asked to give additional blood samples so as laboratory tests can measure the level of Truvada in their blood. These tests can tell how regularly a man has been taking his tablets.
Please tick one box per row to show how much you agree or disagree with each of these statements:

	Strongly Agree	Agree	Neutral or uncertain	Disagree	Strongly Disagree
I think it is a good idea to check how regularly men are taking their tablets by measuring the level of Truvada in their blood	☐	☐	☐	☐	☐
Men report how regularly they take their tablets so there is no need to measure the level of Truvada in their blood	☐	☐	☐	☐	☐
When I am on Truvada, I would like to find out the level of Truvada in my blood	☐	☐	☐	☐	☐

6. **Accurately answering questions about sexual behaviour can be difficult as some people forget details or don't want to share all details.** *Thinking about the last 6 months, please tick one box per row to show how much you agree or disagree with each of these statements:*

	Strongly Agree	Agree	Neutral or uncertain	Disagree	Strongly Disagree
When completing the questionnaires, I find it difficult to remember my sexual activity in the last 30 days	☐	☐	☐	☐	☐
When completing the questionnaires, I am able to report my sexual activity honestly	☐	☐	☐	☐	☐
I find the questionnaires make it difficult to be accurate about my sexual activity	☐	☐	☐	☐	☐

7. **How many other men do you know (partners, friends or acquaintances) who have also joined the PROUD study?** *Please provide an estimate if you do not know the exact number* ☐☐

8. **Since joining the study, how many men that are also in the study, have you had sex with?** *Please provide an estimate if you do not know the exact number* ☐☐

9. **What do you most like about the study?**

10. **What do you least like about the study?**

ONLY ANSWER THE NEXT QUESTION IF YOU ARE CURRENTLY TAKING TRUVADA PREP AS PART OF THE PROUD STUDY

11. Accurately answering questions about taking PrEP can be difficult as some people forget details or don't want to report not taking tablets. *Thinking about the last 6 months, please tick one box per row to show how much you agree or disagree with each of these statements:*

	Strongly Agree	Agree	Neutral or uncertain	Disagree	Strongly Disagree
When completing the questionnaires, I find it difficult to remember when I have taken the tablets in the last 30 days	☐	☐	☐	☐	☐
When completing the questionnaires, I feel confident about saying when I have not taken tablets	☐	☐	☐	☐	☐
I find it difficult to report when I took my tablets on the monthly questionnaire	☐	☐	☐	☐	☐
I find it easy to report when I took my tablets on the daily diary	☐	☐	☐	☐	☐

Thank you for finishing the questionnaire.

Please place in the provided envelope and hand to a <u>doctor or nurse</u>
