# Supplementary material for: Acceptability of an open-label wait-listed trial design: Experiences from the PROUD PrEP study
Source: PLoS One. 2017 Apr 20;12(4):e0175596. doi: 10.1371/journal.pone.0175596 (PMC5398545; doi:10.1371/journal.pone.0175596)
Supplement: S1 Table — (DOCX) [file pone.0175596.s003.docx]

| **S1 Table: Employment and place of birth data tables** | | |  |  |  |  |
| --- | --- | --- | --- | --- | --- | --- |
|  |  |  |  |  |  |  |
| **Acceptability of the study design** |  |  |  |  |  |  |
|  | **Employment** | | | **Place of birth** | | |
|  | **Un- employed** | **Employed** | **P-value** | **Outside UK** | **In UK** | **P-value** |
|  | **73 (18%)** | **339 (82%)** |  | **169 (41%)** | **243 (59%)** |  |
| **Here are some statements that men might say about the PROUD study.** |  |  |  |  |  |  |
| **I am glad I joined the PROUD study** |  |  | 0.755 |  |  | 0.628 |
| Agree | 72 (99%) | 322 (96%) |  | 159 (96%) | 235 (97%) |  |
|  | 98.63 | 96.41 |  | 96.36 | 97.11 |  |
| neutral/uncertain | 1 (1%) | 11 (3%) |  | 5 (3%) | 7 (3%) |  |
|  | 1.37 | 3.29 |  | 3.03 | 2.89 |  |
| Disagree | 0 (0%) | 1 (1%) |  | 1 (1%) | 0 (0%) |  |
|  | 0 | 0.3 |  | 0.61 | 0 |  |
| **I expect to stay in the study for the whole 2 years** |  |  | 0.273 |  |  | 0.710 |
| Agree | 69 (94%) | 320 (96%) |  | 156 (95%) | 233 (96%) |  |
|  | 94.52 | 95.81 |  | 94.55 | 96.28 |  |
| neutral/uncertain | 2 (3%) | 12 (3%) |  | 7 (4%) | 7 (3%) |  |
|  | 2.74 | 3.59 |  | 4.24 | 2.89 |  |
| Disagree | 2 (3%) | 2 (1%) |  | 2 (1%) | 2 (1%) |  |
|  | 2.74 | 0.6 |  | 1.21 | 0.83 |  |
| **I would like to join another PrEP study after this** |  |  | 0.410 |  |  | 0.660 |
| Agree | 65 (89%) | 288 (86%) |  | 143 (87%) | 210 (87%) |  |
|  | 89.04 | 86.23 |  | 86.67 | 86.78 |  |
| neutral/uncertain | 6 (8%) | 41 (12%) |  | 18 (11%) | 29 (12%) |  |
|  | 8.22 | 12.28 |  | 10.91 | 11.98 |  |
| Disagree | 2 (3%) | 5 (2%) |  | 4 (2%) | 3 (1%) |  |
|  | 2.74 | 1.5 |  | 2.42 | 1.24 |  |
| **I think the chance of being in the deferred group and not getting Truvada for a year might put other men off joining the study** |  |  | 0.764 |  |  | 0.533 |
| Agree | 55 (75%) | 243 (74%) |  | 117 (73%) | 181 (75%) |  |
|  | 75.34 | 73.86 |  | 72.67 | 75.1 |  |
| neutral/uncertain | 13 (18%) | 54 (16%) |  | 26 (16%) | 41 (17%) |  |
|  | 17.81 | 16.41 |  | 16.15 | 17.01 |  |
| Disagree | 5 (7%) | 32 (10%) |  | 18 (11%) | 19 (8%) |  |
|  | 6.85 | 9.73 |  | 11.18 | 7.88 |  |
| **The written information I was given clearly explained the study** |  |  | 0.168 |  |  | 1.000 |
| Agree | 70 (96%) | 326 (98%) |  | 160 (98%) | 236 (97%) |  |
|  | 95.89 | 97.9 |  | 97.56 | 97.52 |  |
| neutral/uncertain | 2 (3%) | 7 (2%) |  | 4 (2%) | 5 (2%) |  |
|  | 2.74 | 2.1 |  | 2.44 | 2.07 |  |
| Disagree | 1 (1%) | 0 (0%) |  | 0 (0%) | 1 (1%) |  |
|  | 1.37 | 0 |  | 0 | 0.41 |  |
|  |  |  |  |  |  |  |

| **Acceptability of the study visits** |  |  |  |  |  |  |
| --- | --- | --- | --- | --- | --- | --- |
|  | **Employment** | | | **Place of birth** | | |
|  | **Un- employed** | **Employed** | **P-value** | **Outside UK** | **In UK** | **P-value** |
|  | **73 (18%)** | **339 (82%)** |  | **169 (41%)** | **243 (59%)** |  |
| **Here are some statements that men might say about the PROUD study.** |  |  |  |  |  |  |
| **Visiting the clinic every 3 months is not a problem** |  |  | 0.944 |  |  | 0.503 |
| Agree | 65 (89%) | 298 (90%) |  | 151 (92%) | 213 (88%) |  |
|  | 89.04 | 89.49 |  | 92.07 | 88.02 |  |
| neutral/uncertain | 6 (8%) | 27 (8%) |  | 10 (6%) | 22 (9%) |  |
|  | 8.22 | 8.11 |  | 6.1 | 9.09 |  |
| Disagree | 2 (3%) | 8 (2%) |  | 3 (2%) | 7 (3%) |  |
|  | 2.74 | 2.4 |  | 1.83 | 2.89 |  |
| **I like having regular HIV tests** |  |  | 0.383 |  |  | 0.268 |
| Agree | 66 (92%) | 311 (93%) |  | 158 (96%) | 219 (91%) |  |
|  | 91.67 | 93.39 |  | 95.76 | 91.25 |  |
| neutral/uncertain | 6 (8%) | 17 (5%) |  | 6 (3.5%) | 17 (7%) |  |
|  | 8.33 | 5.11 |  | 3.64 | 7.08 |  |
| Disagree | 0 (0%) | 5 (2%) |  | 1 (0.5%) | 4 (2%) |  |
|  | 0 | 1.5 |  | 0.61 | 1.67 |  |
| **I do not like having regular STI tests** |  |  | 0.171 |  |  | 0.560 |
| Agree | 2 (3%) | 23 (7%) |  | 12 (7%) | 13 (5%) |  |
|  | 2.74 | 6.91 |  | 7.27 | 5.39 |  |
| neutral/uncertain | 8 (11%) | 20 (6%) |  | 9 (6%) | 18 (8%) |  |
|  | 10.96 | 6.01 |  | 5.45 | 7.47 |  |
| Disagree | 63 (86%) | 290 (87%) |  | 144 (87%) | 210 (87%) |  |
|  | 86.3 | 87.09 |  | 87.27 | 87.14 |  |
| **I am able to access as much support to reduce my risk of HIV and STIs as I need** |  |  | 0.383 |  |  | 0.094 |
| Agree | 63 (86%) | 302 (91%) |  | 153 (94%) | 213 (88%) |  |
|  | 86.3 | 90.96 |  | 93.87 | 88.02 |  |
| neutral/uncertain | 9 (12.5%) | 26 (8%) |  | 8 (5%) | 26 (11%) |  |
|  | 12.33 | 7.83 |  | 4.91 | 10.74 |  |
| Disagree | 1 (1.5%) | 4 (1%) |  | 2 (1%) | 3 (1%) |  |
|  | 1.37 | 1.2 |  | 1.23 | 1.24 |  |
| **Some men have been, or will be, asked to give additional blood samples so as laboratory tests can measure the level of Truvada in their blood. These tests can tell how regularly a** |  |  |  |  |  |  |
| **I think it is a good idea to check how regularly men are taking their tablets by measuring the level of Truvada in their blood** |  |  | 0.657 |  |  | 0.400 |
| Agree | 69 (95%) | 320 (96%) |  | 157 (95%) | 232 (96%) |  |
|  | 94.52 | 96.1 |  | 95.15 | 96.27 |  |
| neutral/uncertain | 4 (5%) | 11 (3%) |  | 8 (5%) | 7 (3%) |  |
|  | 5.48 | 3.3 |  | 4.85 | 2.9 |  |
| Disagree | 0 (0%) | 2 (1%) |  | 0 (0%) | 2 (1%) |  |
|  | 0 | 0.6 |  | 0 | 0.83 |  |
| **Men report how regularly they take their tablets so there is no need to measure the level of Truvada in their blood** |  |  | 0.556 |  |  | 0.580 |
| Agree | 11 (15%) | 53 (16%) |  | 28 (17%) | 35 (15%) |  |
|  | 15.07 | 15.92 |  | 16.97 | 14.52 |  |
| neutral/uncertain | 25 (34%) | 93 (28%) |  | 51 (31%) | 68 (28%) |  |
|  | 34.25 | 27.93 |  | 30.91 | 28.22 |  |
| Disagree | 37 (51%) | 187 (56%) |  | 86 (52%) | 138 (57%) |  |
|  | 50.68 | 56.16 |  | 52.12 | 57.26 |  |
| **When I am on Truvada, I would like to find out the level of Truvada in my blood** |  |  | 0.433 |  |  | 0.397 |
| Agree | 61 (84%) | 286 (86%) |  | 145 (88%) | 202 (84%) |  |
|  | 83.56 | 85.89 |  | 87.88 | 83.82 |  |
| neutral/uncertain | 12 (16%) | 41 (12%) |  | 19 (11%) | 34 (14%) |  |
|  | 16.44 | 12.31 |  | 11.52 | 14.11 |  |
| Disagree | 0 (0%) | 6 (2%) |  | 1 (1%) | 5 (2%) |  |
|  | 0 | 1.8 |  | 0.61 | 2.07 |  |
|  |  |  |  |  |  |  |

| **Acceptability of the study data collection** | |  |  |  |  |  |
| --- | --- | --- | --- | --- | --- | --- |
|  | **Employment** | | | **Place of birth** | | |
|  | **Un- employed** | **Employed** | **P-value** | **Outside UK** | **In UK** | **P-value** |
|  | **73 (18%)** | **339 (82%)** |  | **169 (41%)** | **243 (59%)** |  |
| **Here are some statements that men might say about the PROUD study.** |  |  |  |  |  |  |
| **I don’t mind completing the monthly sexual behaviour questionnaires** |  |  | 0.738 |  |  | 0.785 |
| Agree | 59 (82%) | 275 (82%) |  | 136 (82%) | 198 (82%) |  |
|  | 81.94 | 82.34 |  | 82.42 | 82.16 |  |
| neutral/uncertain | 11 (15%) | 43 (13%) |  | 23 (14%) | 31 (13%) |  |
|  | 15.28 | 12.87 |  | 13.94 | 12.86 |  |
| Disagree | 2 (3%) | 16 (5%) |  | 6 (4%) | 12 (5%) |  |
|  | 2.78 | 4.79 |  | 3.64 | 4.98 |  |
| **I dislike completing the sexual behaviour diary** |  |  | 0.064 |  |  | 0.911 |
| Agree | 13 (18%) | 64 (19%) |  | 31 (19%) | 46 (19%) |  |
|  | 17.81 | 19.34 |  | 19.02 | 19.09 |  |
| neutral/uncertain | 26 (36%) | 75 (23%) |  | 39 (24%) | 62 (26%) |  |
|  | 35.62 | 22.66 |  | 23.93 | 25.73 |  |
| Disagree | 34 (46%) | 192 (58%) |  | 93 (57%) | 133 (55%) |  |
|  | 46.58 | 58.01 |  | 57.06 | 55.19 |  |
| **I like completing the questionnaires on-line** |  |  | 0.594 |  |  | 0.183 |
| Agree | 37 (51%) | 185 (57%) |  | 88 (54%) | 135 (57%) |  |
|  | 51.39 | 56.57 |  | 54.32 | 56.96 |  |
| neutral/uncertain | 26 (36%) | 98 (30%) |  | 57 (35%) | 66 (28%) |  |
|  | 36.11 | 29.97 |  | 35.19 | 27.85 |  |
| Disagree | 9 (13%) | 44 (13%) |  | 17 (11%) | 36 (15%) |  |
|  | 12.5 | 13.46 |  | 10.49 | 15.19 |  |
| **I would prefer to answer the sexual behaviour questions on a mobile device** |  |  | 0.074 |  |  | 0.316 |
| Agree | 34 (47%) | 202 (61%) |  | 103 (62%) | 133 (55%) |  |
|  | 46.58 | 60.48 |  | 62.42 | 54.96 |  |
| neutral/uncertain | 27 (37%) | 98 (29%) |  | 46 (28%) | 79 (33%) |  |
|  | 36.99 | 29.34 |  | 27.88 | 32.64 |  |
| Disagree | 12 (16%) | 34 (10%) |  | 16 (10%) | 30 (12%) |  |
|  | 16.44 | 10.18 |  | 9.7 | 12.4 |  |
| **Accurately answering questions about sexual behaviour can be difficult as some people forget details or don’t want to share all details.** |  |  |  |  |  |  |
| **When completing the questionnaires, I am able to report my sexual activity honestly** |  |  | 0.840 |  |  | 0.758 |
| Agree | 61 (87%) | 291 (88%) |  | 143 (87%) | 209 (89%) |  |
|  | 87.14 | 88.18 |  | 86.67 | 88.94 |  |
| neutral/uncertain | 7 (10%) | 31 (9%) |  | 17 (10%) | 21 (9%) |  |
|  | 10 | 9.39 |  | 10.3 | 8.94 |  |
| Disagree | 2 (3%) | 8 (3%) |  | 5 (3%) | 5 (2%) |  |
|  | 2.86 | 2.42 |  | 3.03 | 2.13 |  |
| **When completing the questionnaires, I find it difficult to remember my sexual activity in the last 30 days** |  |  | 0.278 |  |  | 0.190 |
| Agree | 33 (47%) | 155 (47%) |  | 78 (47%) | 110 (47%) |  |
|  | 47.14 | 46.97 |  | 47.27 | 46.81 |  |
| neutral/uncertain | 16 (23%) | 52 (16%) |  | 34 (21%) | 34 (14%) |  |
|  | 22.86 | 15.76 |  | 20.61 | 14.47 |  |
| Disagree | 21 (30%) | 123 (37%) |  | 53 (32%) | 91 (39%) |  |
|  | 30 | 37.27 |  | 32.12 | 38.72 |  |
| **I find the questionnaires make it difficult to be accurate about my sexual activity** |  |  | 0.349 |  |  | 0.332 |
| Agree | 21 (30%) | 92 (28%) |  | 42 (25%) | 71 (30%) |  |
|  | 30 | 27.88 |  | 25.45 | 30.21 |  |
| neutral/uncertain | 23 (33%) | 86 (26%) |  | 51 (31%) | 58 (25%) |  |
|  | 32.86 | 26.06 |  | 30.91 | 24.68 |  |
| Disagree | 26 (37%) | 152 (46%) |  | 72 (44%) | 106 (45%) |  |
|  | 37.14 | 46.06 |  | 43.64 | 45.11 |  |
|  |  |  |  |  |  |  |
|  |  |  |  |  |  |  |
